# A Classic Cornelia De Lange Syndrome Type 5 (CdLS5) With a De Novo Missense Variation of p.Gly210Arg in the HDAC8 Gene With a Novel Phenotype of Generalized Dystonia

**DOI:** 10.7759/cureus.60838

**Published:** 2024-05-22

**Authors:** Monika Chhajed, Meenakshi Lallar, Pradeep Kumar Gunasekaran, Amit Jain, Lokesh Saini

**Affiliations:** 1 Pediatric Neurology, Chaitanya Hospital, Chandigarh, IND; 2 Clinical Genetics, Prime Diagnostics, Chandigarh, IND; 3 Pediatrics, All India Institute of Medical Sciences, Jodhpur, IND; 4 Radiodiagnosis, Maharishi Markandeshwar Institute of Medical Sciences and Research, Ambala, IND

**Keywords:** cdls5, cdls, dystonias, hdac8 gene, cornelia de lange syndrome

## Abstract

Cornelia de Lange syndrome (CdLS) is a rare neurodevelopmental disorder characterized by distinct dysmorphic facies, skeletal anomalies, and failure to thrive. CdLS type 5 (CdLS5) is caused by the HDAC8 gene mutations on chromosome Xq13.1 with X-linked dominant inheritance. We report our observation of an individual with CdLS5 with de novo missense mutation presenting with a novel phenotype of generalized dystonia. A four-month-old girl, second born to a non-consanguineous couple, presented with developmental delay, failure to thrive, and spastic quadriparesis. She had a history of intrauterine growth retardation in the third trimester of pregnancy. Facial gestalt was suggestive of CdLS. She had marked axial and appendicular dystonia. A skeletal survey and magnetic resonance imaging (MRI) with magnetic resonance spectroscopy (MRS) brain studies were normal. Genetic testing revealed a heterozygous missense variation c.628G>C in the HDAC8 gene. She was treated with trihexyphenidyl and clonazepam, followed by syndopa. On follow-up assessment at 22 months of age, the dystonia gradually improved but not entirely over time with medication. It is already known that single gene disorders, including SCN1A, SCN2A, KCNQ2, PRRT2, and pyridoxine deficiency, can result in isolated dystonia; we add CdLS5 (HDAC8 variation) to this expanding spectrum.

## Introduction

Cornelia de Lange syndrome (CdLS) is a clinically variable and genetically heterogeneous, rare neurodevelopmental disorder first described as "typus degenerativus Amstelodamensis" (Amsterdam degeneration type) by a Dutch Pediatrician and Neuropathologist, Cornelia Catharina de Lange in 1933 [1]. CdLS is characterized by distinct dysmorphic facies, skeletal anomalies, and failure to thrive. Individuals with CdLS usually have pathogenic variants in genes codifying for the cohesin complex like NIPBL (Nipped-B-like protein), SMC1A, SMC3, RAD21, and HDAC8 [2,3]. The additional genes recently described are BRD4, ANKRD11, and MAU2 [2]. The incidence of CdLS is reported at one per 10,000-50,000 newborns [4]. CdLS type 5 (CdLS5), a multisystemic genetic disorder, is caused by the HDAC8 (histone deacetylase 8) gene mutations (MIM*300269) on chromosome Xq13.1 with X-linked dominant inheritance [4,5]. HDAC8 encodes the lysine deacetylase for the SMC3 (structural maintenance of chromosomes 3) subunit of cohesion, which is involved in the release of cohesion complex [2,5]. HDAC8 mutations affect the cohesin acetylation cycle by the loss of HDAC8 activity, resulting in heightened acetylation of SMC3 and ineffective dissolution of the "used" cohesin complex [5]. The common features of CdLS5 similar to typical CdLS are postnatal growth retardation, microcephaly, developmental delay, facial dysmorphism including hooding of eyelids, arched eyebrows, depressed nasal bridge, micrognathia, cleft palate, long philtrum, gastroesophageal reflux disease (GERD), congenital heart disease, limb defects (more in upper limbs), and genitourinary abnormalities [2,6-9]. The other features reported in CdLS5 patients are oligodontia, myopia, and atrial septal defect [8]. The phenotype of CdLS5 presents with a spectrum of clinical symptoms without characteristic facial features of CdLS [6,7]. The common differential diagnoses are Coffin-Siris syndrome, Wiedemann-Steiner syndrome, and Rubinstein-Taybi syndrome. We describe a de novo missense mutation in the HDAC8 gene and expand the phenotype of the HDAC8 gene associated with CdLS5 to include generalized dystonia.

## Case presentation

A four-month-old girl, second born to a non-consanguineous couple, presented with developmental delay, failure to thrive, abnormal twisting postures suggestive of dystonia, and spastic quadriparesis. The developmental delay was predominant in the language and motor domains. She had a history of intrauterine growth retardation in the third trimester of pregnancy. She was born prematurely at 34 weeks, with a birth weight of 1.95 kg, and had respiratory distress and feeding issues, but there was no history of neonatal seizures or hypoglycemia. The family history was unremarkable. Around four months of age, parents noticed tightness of limbs and body and failure to thrive.

Physical examination

**Figure 1:**
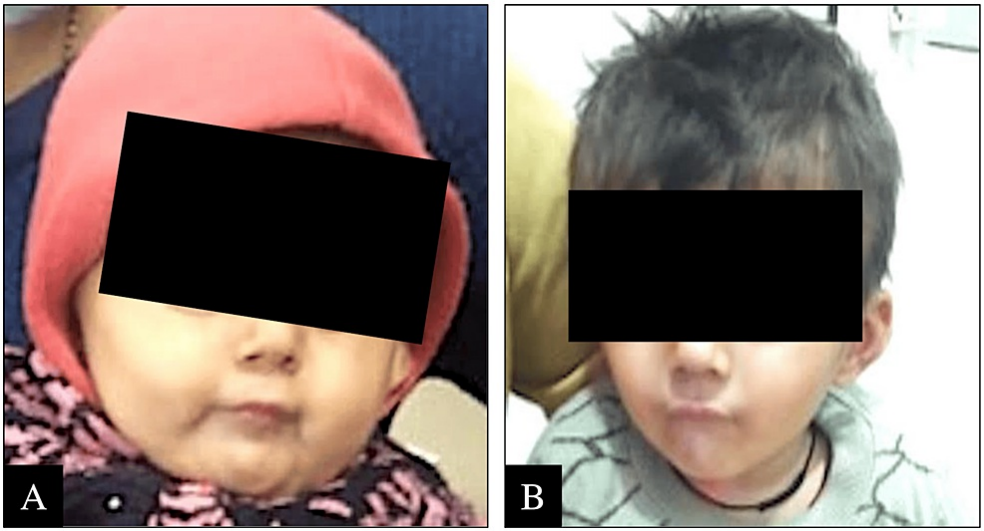
Facial CdLS Gestalt in the Index Child (A) the index child at the age of 11 months; (B) the index child at the age of 22 months CdLS, Cornelia de Lange syndrome

On evaluation, there was a global developmental delay; weight was 5.1 kg (3rd-15th centile; WHO child growth standards), length was 55 cm (<3rd centile), and head circumference was 38.5 cm (3rd-15th centile). Facial gestalt was suggestive of CdLS (Figure 1A). The dysmorphisms noted were brachycephaly, flattened face, arched eyebrows, deep-set eyes, hypertelorism, long philtrum, broad root of nose, and depressed broad nasal bridge. She had no limb anomalies or hirsutism. She had marked axial and appendicular dystonia. Fundus examination was normal. Clinical scoring for diagnosing CdLS using cardinal and suggestive features was suggestive of classic CdLS [4].

Investigations

Cerebrospinal fluid (CSF) examination showed two cells, glucose of 66 mg/dL, and protein of 32.2 mg/dL. Tandem mass spectrometry (TMS)/gas chromatography-mass spectrometry (GC-MS) study was normal. The skeletal survey was within normal limits. Brain-evoked auditory response (BERA) testing was normal. Magnetic resonance imaging (MRI) with magnetic resonance spectroscopy (MRS) of the brain was normal. Targeted gene sequencing of known OMIM genes was done by selective capture and sequencing of the protein-coding regions, exons, splice site, and other clinical relevance in the genome. Gene capture was done using a relevant TWIST CEV4 custom capture kit. The libraries were sequenced to mean >80-100X coverage on the Illumina sequencing platform. The sequences obtained were aligned to the human reference genome (GRCh38.p13) using the BWA aligner. The germline variants identified in the sample were deeply annotated using the VariMAT pipeline. Clinically relevant mutations in both coding and non-coding regions were annotated using published variants in literature and a set of disease databases - ClinVar, OMIM, HGMD, LOVD, DECIPHER (population CNV), and SwissVar. Common variants were filtered based on allele frequency in various populations and internal databases. The non-synonymous variant’s effect was calculated using multiple algorithms such as PolyPhen-2, SIFT, MutationTaster2, and LRT [10]. Clinically significant variants were used for interpretation and reporting. The results were classified as per the American College of Medical Genetics (ACMG) guidelines [11]. A heterozygous missense variation was detected, HDAC8, c.628G>C (p.Gly210Arg)(ENST00000373573.9). Sanger sequencing was done in parents and detected the wild-type allele, confirming de-novo heterozygous HDAC8, c.628G>C in the proband.

Outcome and follow-up

She was treated with trihexyphenidyl and clonazepam, followed by syndopa for dystonia management. She had GERD and was treated with thickening of feeds, lower-volume feedings, and after-feeding positioning. On follow-up assessment at 22 months of age, her weight was 6.6 kg (<3rd centile), length was 77 cm (<3rd centile), and head circumference was 45.5 cm (3rd-15th centile) (Figure 1B). The dystonia gradually improved but not entirely over time with medication. Clonazepam was tapered and stopped, and the syndopa dose was reduced. She was managed with GERD therapy for feeding issues.

## Discussion

CdLS5 (MIM#300882) is caused by HDAC8 gene mutations (MIM*300269) on chromosome Xq13.1 with X-linked dominant inheritance [2,4,5]. Individuals with HDAC8-related CdLS have a delayed anterior fontanelle closure and more pronounced ocular hypertelorism, hooding of the eyelids, a broader nose, and dental anomalies. The key phenotype in the index child here is similar to the NIPBL (Nipped-B-like protein) gene associated with classic CdLS, including growth retardation and gastroesophageal reflux [3]. However, microcephaly, cardiac, and genitourinary abnormalities were not seen. The index child had similar features of dysmorphism seen in CdLS5 patients, including arched eyebrows, depressed nasal bridge, hypertelorism, and long philtrum [2,6-9]. The index child had no limb anomalies, a characteristic frequently observed in CdLS5 patients [7]. The phenotype can also be more variable because HDAC8-associated CdLS is X-linked and influenced by random X-inactivation in females [6].

Jezela-Stanek et al. reported a case of CdLS5 with myopia, bilateral conductive hearing loss, short philtrum, atrial septal defect, gastroesophageal reflux, and posterior urethral valves [8]. Dystonia has not yet been reported in CdLS patients. We did not find any other underlying etiology for dystonia in investigations. HDAC8-related CdLS is usually caused by point mutations (missense or null) spread throughout the gene. Depending upon the position of these mutations, they determine the residual enzymatic activity, which in turn leads to varying degrees of loss of acetylation and thus contributes to the variable phenotype in patients [2,6]. The index child’s de novo missense variant c.628G>C is located in the loop region of the HDAC8 protein surrounding the active site [12]. This loop region is critical in protein-protein interactions or conferring different substrate specificities [13]. The missense mutations cause the loss of the enzymatic function of HDAC8 [2,6].

As in our patient, the management is primarily symptomatic and consists of speech and vocational therapies. The feeding issues can be challenging, and treatment with thickening of feeds, lower-volume feedings, after-feeding positioning, antacids, and nasogastric tubes may be required. Gastrostomy can be taken up on a case-to-case basis under the guidance of a pediatric gastroenterologist. The short stature can be treated with recombinant human growth hormone (rhGH). The index child is currently managed by a multidisciplinary team in adherence to the management guidelines [4]. Since it is already known that single gene disorders, including SCN1A, SCN2A, KCNQ2, PRRT2, and pyridoxine deficiency, can result in isolated dystonia, we add CdLS5 (HDAC8 variation) to this expanding spectrum. The limitation of this report is that the follow-up is short-term in the index child.

## Conclusions

We report a CdLS5 with de novo missense mutation c.628G>C in the HDAC8 gene and a novel phenotype of generalized dystonia, further delineating the phenotypic spectrum. The dystonia responded to regular medication and has waned significantly but not entirely over time. Prospective follow-up of this patient will help us further elucidate the association between the HDAC8 gene and dystonias.
